# Suppressed Plastic Anisotropy via Sigma-Phase Precipitation in CoCrFeMnNi High-Entropy Alloys

**DOI:** 10.3390/ma17061265

**Published:** 2024-03-08

**Authors:** Tae Hyeong Kim, Jaimyun Jung, Jae Wung Bae

**Affiliations:** 1 Department of Metallurgical Engineering, Pukyong National University, Busan 48513, Republic of Korea; 2 Department of Materials AI & Big-Data, Korea Institute of Materials Science (KIMS), Changwon 51508, Republic of Korea

**Keywords:** high-entropy alloys, precipitates, plastic anisotropy, visco-plastic self-consistent model

## Abstract

The effect of sigma-phase precipitation on plastic anisotropy of the equiatomic CoCrFeMnNi high-entropy alloy was investigated. Annealing at 700 °C after cold-rolling leads to the formation of the Cr-rich σ phase with a fraction of 2.7%. It is experimentally found that the planar anisotropy (∆*r* = −0.16) of the CoCrFeMnNi alloy annealed at 700 °C is two times lower than that of the alloy annealed at 800 °C (∆*r* = −0.35). This observation was further supported by measuring the earing profile of cup specimens after the deep drawing process. The plastic strain ratio, normal anisotropy, and planar anisotropy were also predicted using the visco-plastic self-consistent model. The results indirectly indicated that the reduction of plastic anisotropy in alloy annealed at 700 °C can be attributed to the formation of the *σ* phase.

## 1. Introduction

High-entropy alloys (HEAs), defined as multicomponent alloys composed of at least five principal elements in an equiatomic or near-equiatomic composition, have been attracting considerable attention as promising materials in the material science engineering community due to their unique alloy design strategy, microstructures, and physical properties [1,2,3,4]. To further enhance the mechanical properties of HEAs, research trends on HEAs with entropy-based alloy design for simple solid solution structures have widely expanded to comprise various strengthening strategies, including phase-metastability, grain-refinement, precipitation, segregation, and short-range ordering [5,6,7,8,9]. Second-phase precipitation was initially regarded as a counter-intuitive feature of the original design concept of HEAs. However, recent studies have shown that precipitation strengthening can effectively improve the properties of face-centered cubic (FCC) HEAs. Notably, some studies utilizing the brittle intermetallic sigma (*σ*) phase, the formation of which was considered undesirable in the early studies on HEAs, as a strengthener have been reported [10,11].

Many structural alloys having significant potential with a combination of high strength and ductility, often have major drawbacks in poor formability that postpone their commercialization. The formability of sheet metals is closely related to the tensile properties such as strain-hardening exponent (*n*), strain-rate sensitivity (*m*), and normal and planar anisotropy (mean *r* and ∆*r* values, respectively) [12]. In particular, the importance of plastic anisotropy has been recognized for many years. It causes earing in drawn cups and thereby creates additional scraps in sheet metal forming or delayed fracture after formation [13]. Investigations have demonstrated that the anisotropic behavior is closely related to ∆*r* value and results primarily from the presence of crystallographic texture, grain morphology, and the development of slip systems [14]. The formation of crystallographic texture in an annealed recrystallized state is significantly influenced by the deformation texture, which in turn is closely related to the stacking fault energy (SFE) of the materials [15]. Equiatomic CoCrFeMnNi HEA is known to have low SFE, which is also supported by the formation of deformation twinning and planar array of dislocations during plastic deformation [16]. CoCrFeMnNi HEA with low SFE has a similar recrystallization texture to that of metals with low stacking fault energy such as twinning-induced plasticity (TWIP) steels [15]. Bae et al. [17] first investigated the deep drawing behavior of the hot-rolled and subsequently annealed coarse-grained CoCrFeMnNi HEA. Based on his report, the hot-rolled and annealed alloy exhibited negligible planar anisotropy of ∆*r* (0.06) and earing behavior. However, cold-rolling can cause a stronger texture with elongated grains along the rolling direction, and the material becomes anisotropic even after recrystallization annealing. It was reported that the recrystallization texture after cold rolling in the CoCrFeMnNi HEA is composed of {001}<100> Cube component, which is an important texture component affecting earing behavior [18]. It is thus expected that CoCrFeMnNi HEA, when cold-rolled and subsequently annealed, also exhibits anisotropic properties by the presence of preferred orientation.

Meanwhile, the slip activities within the matrix are greatly affected depending on whether or not slips occur adjacent to the precipitates [14]. The presence of precipitates can influence grain rotations such that the slip activity differs significantly compared to the case when no precipitate is present. However, previous studies have focused on the utilization of second-phase precipitates as strengtheners, and very limited research has been carried out on the investigation of formability of HEAs, which is critical for manufacturing and industrial applications. Plastic anisotropy is recognized as one of the measures of sheet metal formability, significantly influenced by crystallographic texture. Material anisotropy can be determined by three Lankford coefficients: *r*_0_, *r*_45_, and *r*_90_ obtained in uniaxial tensile tests along the rolling direction, at 45°, and at 90° from the rolling direction, respectively. The effect of mechanical anisotropy leads to the formation of ears and troughs around the deep-drawn cup. To achieve a flange with a flat top edge, the ears must be trimmed to the lowest level after the drawing process, necessitating additional trimming process and resulting in material loss. Hence, controlling texture and microstructure during processing is crucial for minimizing such losses and enhancing formability. Motivated by this, and deviating from recent research trends on the role of second-phase precipitation in strengthening, we explore for the first time in the field of HEAs the effect of *σ* phase formation on plastic anisotropy in the CoCrFeMnNi HEA. The results indicate that plastic anisotropy is mitigated by the formation of the *σ* phase. Our findings demonstrate that, although *σ* phase formation is generally avoided in typical HEAs due to its adverse effects on ductility, it can be strategically used in the design of HEAs to achieve a desirable combination of strength, ductility, and formability.

## 2. Materials and Methods

The equiatomic CoCrFeMnNi HEA was produced using vacuum-induction melting and homogenized at 1100 °C for 6 h under argon gas. The ingots were hot-rolled with a thickness reduction of ~98% in the temperature range of 1020–1100 °C and then cold-rolled at room temperature with ~50% reduction in thickness. The obtained cold-rolled sheets at 1.5 mm thick were annealed at 700 °C and 800 °C for 1 h in a furnace followed by water quenching. For convenience, the specimens annealed at 700 °C and 800 °C are referred to as A700 and A800, respectively.

Microstructure characterization was carried out on the annealed samples. Phase constitution was characterized by X-ray diffraction (XRD) with Cu Kα radiation (wavelength = 1.5418 Å) with a step size of 0.04° and scan speed of 2°/min and scanning electron microscope (SEM) imaging with back-scattered electron (BSE). Electron back-scatter diffraction (EBSD) analysis was conducted using a field emission SEM (FE-SEM, XL-30S FEG, FEI Company, Hillsboro, OR, USA). The EBSD data were then interpreted using orientation imaging microscopy (OIM) analysis software (TSL OIM Analysis 5.2). Transmission electron microscopy (TEM, JEM-F200, JEOL Ltd, Tokyo, Japan) analysis was conducted using a JEOL/JEM-F200 equipped with an X-ray energy dispersive spectrometry (EDS) detector at an accelerating voltage of 200 kV. Selected area diffraction patterns (SADPs) were obtained for phase identification.

Uniaxial tension tests were conducted on the specime-type sub-sized tensile specimens (gage length: 6.4 mm, gage width: 2.5 mm, thickness: 1.5 mm) were deformed at a strain rate of 10^−3^ s^−1^ using a universal testing machine (Instron 1361, Instron Corp, Canton, MA, USA) with a vision strain gage system (ARAMIS v6.1, GOM Optical ns cut in the rolling, transverse, and 45-degree directions. The plateMeasuring Techniques, Braunschweig, Germany). The plastic strain ratio (*r*-value: Lankford’s coefficient), normal anisotropy ($\overset{-}{r}$) and planar anisotropy (∆*r*) were determined at a true strain of 0.15 using the following equations:

(1) $$r=-\;\frac{\varepsilon_{w}}{\varepsilon_{w}+\varepsilon_{t}},$$

(2) $$\overset{-}{r}=\frac{r_{0}+2r_{45}+r_{90}}{4},$$

(3) $$r=\frac{r_{0}-2r_{45}+r_{90}}{2},$$

where $\varepsilon_{w}$ and $\varepsilon_{t}$ are the width and thickness strains, and $r_{0}$, $r_{45}$, and $r_{90}$ are the *r* values along 0, 45, and 90 degrees to the rolling direction, respectively. Three samples were tensile-tested for each condition to confirm reproducibility, and representative data are shown.

The deep drawing tests were conducted using a 100-ton double action hydraulic type press machine with circular blanks (diameter 100 mm and thickness 1.5 mm) and beef tallow as a lubricant to reduce the friction between the blank and die. A circular punch (diameter 50 mm) was used, and the drawing ratio (=blank diameter/punch diameter) was 2.0.

The Lankford’s coefficient of the alloy was also predicted using the visco-plastic self-consistent (VPSC) model [19,20]. In the model, which has been used extensively to successfully simulate plastic anisotropy of sheet metals [21,22], each grain within a polycrystalline material is treated as an elliptical visco-plastic inclusion embedded in an anisotropic homogeneous equivalent medium (HEM). In the VPSC model, the strain rate of individual grains can be described in the form of a power law,

(4) $${\dot{\varepsilon }}_{\mathrm{ij}}=\dot{\gamma_{0}}\sum_{s}m_{\mathrm{ij}}^{s}{\left(\frac{m_{\mathrm{kl}}^{s}\sigma_{\mathrm{kl}}}{\tau^{s}}\right)}^{n},$$

where ${\dot{\varepsilon }}_{\mathrm{ij}}$, $\dot{\gamma_{0}}$, $m_{\mathrm{ij}}^{s}$, $\sigma_{\mathrm{kl}}$, $\tau^{s}$, and n represent the strain rate within a grain, reference shear strain rate, the Schmid tensor associated with slip system *s*, the stress in the grain, the threshold stress associated with slip system *s*, and the rate-sensitivity related exponent, respectively. For each grain, the evolution of the threshold stress in slip system *s* due to accumulated shear strain is described by the Voce law as follows

(5) $$\tau^{s}=\tau_{0}^{s}+\left(\tau_{1}^{s}+\theta_{1}^{s}\Gamma \right)\left(1\;-\exp\left(-\Gamma \left|\frac{\theta_{0}^{s}}{\tau_{1}^{s}}\right|\right)\right),$$

where $\tau_{0}^{s}$, ($\tau_{0}^{s}+\tau_{1}^{s}$), $\theta_{0}^{s}$, $\theta_{1}^{s}$, and Γ are the initial critically resolved shear stress, the back-extrapolated critically resolved shear stress, initial hardening rate, the asymptotic hardening rate, and the accumulated shear strain for all slip systems, respectively.

In this work, the initial textures of both A700 and A800 specimens were extracted from their respective EBSD data. To fit the stress–strain curves, the hardening parameters $\tau_{0}^{s}$, $\tau_{1}^{s}$, $\theta_{0}^{s}$, $\theta_{1}^{s}$ for A700 and A800 were set to 195, 280, 390, 22, and 118, 265, 480, 2, respectively.

## 3. Results and Discussion

**Figure 1:**
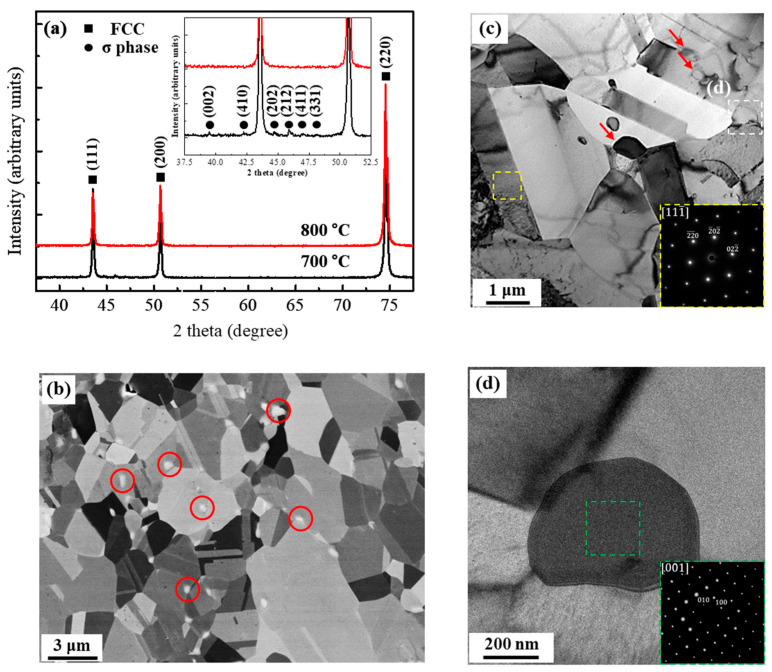
(**a**) X-ray diffraction patterns of A700 and A800 specimens. (**b**) Back-scattered electron (BSE) image of A700 specimen indicating the presence of *σ* phase, as marked by red circles. TEM image of (**c**) FCC matrix and (**d**) tetragonal *σ* phase in A700 specimen. The insets in the TEM image show the SADPs of (**c**) FCC matrix and (**d**) tetragonal *σ* phase, respectively.

**Figure 2:**
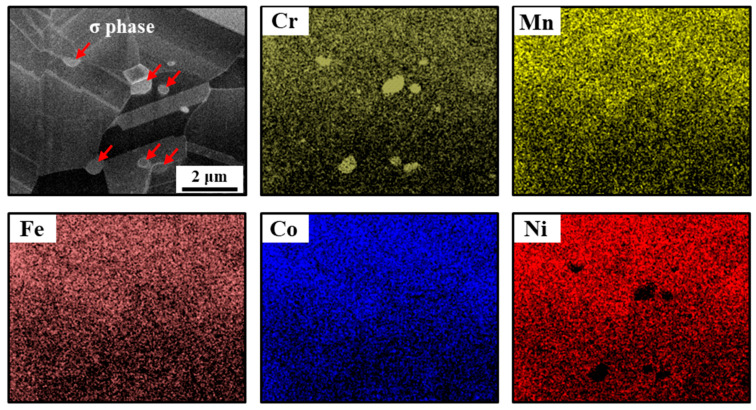
Elemental distribution in A700 specimen measured by TEM-EDS analysis. Arrows in the high-angle annular dark-field image indicate *σ* phase precipitates.

Figure 1a shows XRD patterns of the CoCrFeMnNi alloy after cold rolling and subsequent annealing at 700 °C and 800 °C. The XRD peaks of A800 correspond to the fcc structure with (111), (200), and (220), indicating a single-phase solid solution. On the other hand, the XRD result of the A700 specimen consists of FCC peaks with high intensity and additional peaks. The inset in Figure 1a represents an enlarged range from 37.5° to 52.5°, and several peaks of the second phase in the A700 specimen correspond to *σ* phases with a tetragonal structure [10]. Figure 1b shows an SEM-BSE image of the A700 specimen. Annealing at 700 °C after cold rolling results in not only complete recrystallization but also the formation of submicron *σ* phase particles. The red circles highlighted in the SEM-BSE image indicate the formation of the *σ* phase mostly at the triple junctions of recrystallized grains and within grains. These precipitates exhibit bright contrast in the BSE image. The measured volume fraction and size of *σ* phase particles, determined by means of conventional metallography methods, are 2.7% and 0.35 ± 0.13 μm, respectively. Figure 1c shows the TEM bright-field image of the A700 specimen, which indicates the distribution of fine *σ*-precipitates within the FCC matrix. Inset in Figure 1c is the SADP of the FCC matrix that is taken along the [11$\overset{-}{1}$] zone axes. Figure 1d shows the magnified view obtained from the region marked by the white box in Figure 1c. The inset in Figure 1d is the SADP of the precipitate taken along the [001] zone axes, which confirms that the particle is a tetragonal *σ* phase. Figure 2 shows the elemental mapping distribution of Cr, Mn, Fe, Co, and Ni in the A700 specimen, confirming the Cr enrichment and Ni depletion within the *σ*-precipitates. Additionally, the chemical composition of each phase in A700 was measured by EDS analysis, and the results are summarized in Table 1. The results indicate that the FCC matrix has an overall composition of equiatomic CoCrFeMnNi. On the other hand, Cr is enriched and Ni is depleted in the second phase. This conclusively confirms that the second-phase particles formed in A700 correspond to the *σ* phase with the chemical composition of 19.4Co-43.5Cr-16.3Fe-13.6Mn-7.1Ni, which is approximately consistent with previously reported results [23]. Phase decompositions in CoCrFeMnNi HEA annealed below 800 °C have been reported [24,25], and are attributed to the temperature-dependent stability of the FCC solid solution in CoCrFeMnNi HEA.

**Figure 3:**
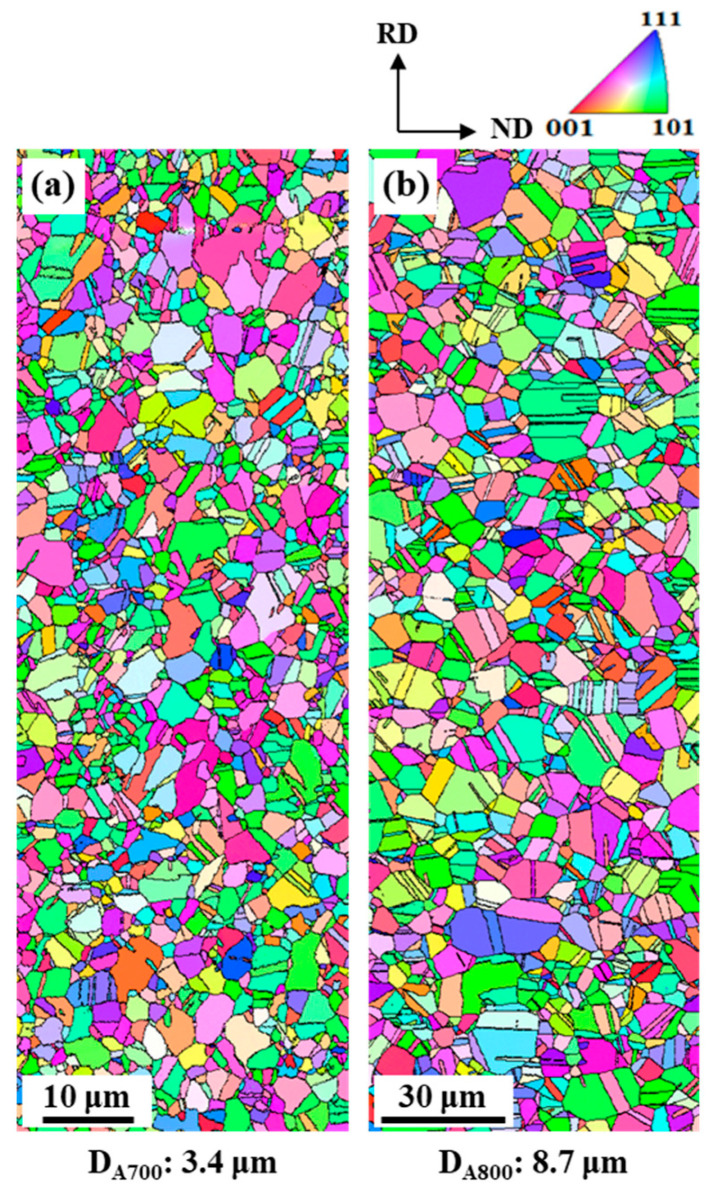
EBSD inverse pole figure (IPF) maps of (**a**) A700 and (**b**) A800 specimens.

The microstructures of both specimens are further confirmed by EBSD analysis (Figure 3a,b), and both images exhibit equiaxed recrystallized grains with numerous annealing twins. The average grain sizes, D, measured from the EBSD images, are 3.4 ± 0.1 μm for the A700 specimen, and 8.7 ± 0.2 μm for the A800 specimen (annealing twin boundaries were not counted). These data, therefore, show that the present CoCrFeMnNi alloy after annealing at temperatures below 800 °C is not a single phase, corresponding to the previous reports [11].

**Figure 4:**
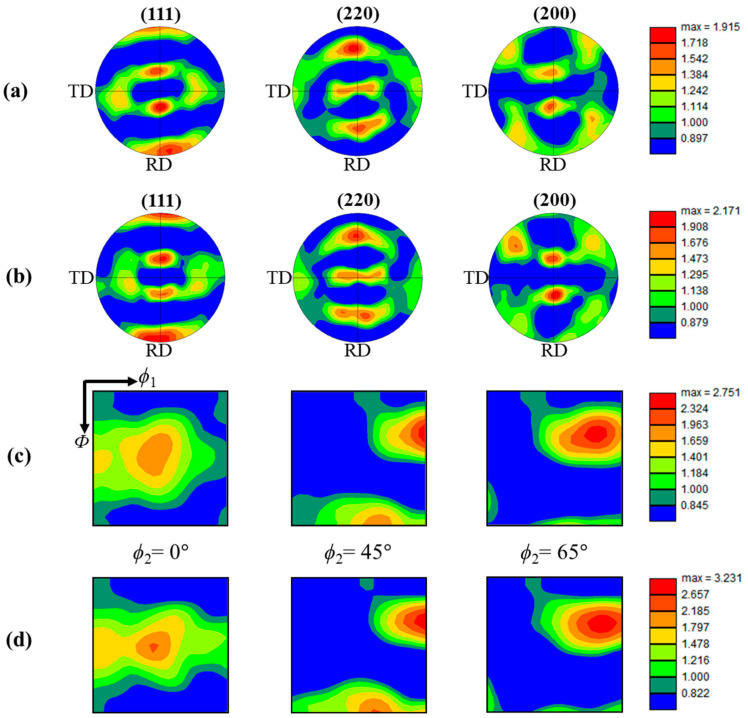
(110), (220), and (200) pole figures of (**a**) A700 and (**b**) A800 specimens. Orientation distribution functions (ODFs) in Euler space of (**c**) A700 and (**d**) A800 specimens.

**Figure 5:**
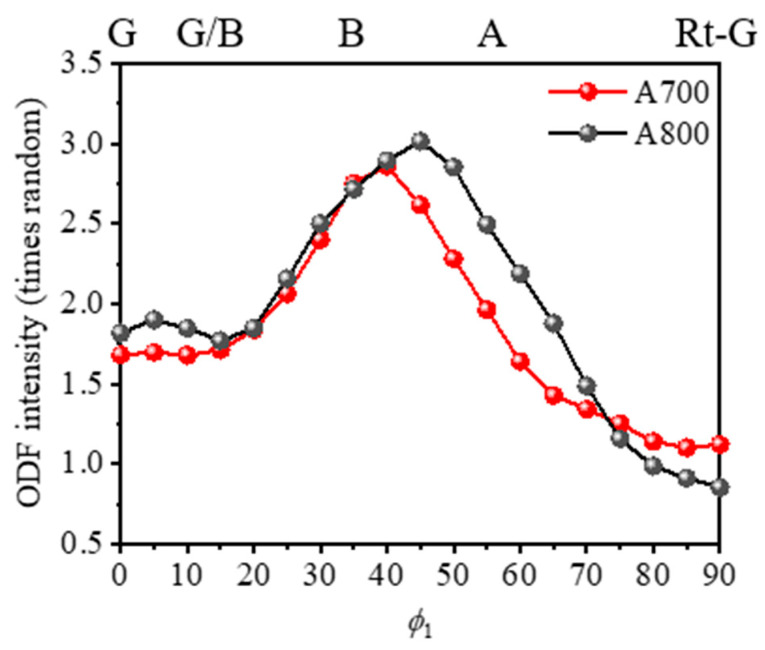
Variation of ODF intensity along *α*-fiber in A700 and A800 specimens.

Figure 4 shows the results of the pole figures (PFs) and orientation distribution function (ODF) sections obtained from the EBSD data in Figure 3a,b. The main texture components in both specimens are similar to each other and could be classified as the orientations of {110}<100> Copper, {123}<634> S, {110}<112> Brass, and {110}<100> Goss, which are known as the typical rolling texture components in FCC metals. We note that, although the specimens were fully recrystallized after cold-rolling and subsequent annealing at 700 and 800 °C, the crystallographic textures of A700 and A800 specimens are composed of the retention of deformation texture formed during cold-rolling. These results indicate the absence of preferential nucleation or growth of specific orientations, which is consistent with previous studies [26]. We further investigated the major texture components along the *α*-fiber in A700 and A800. Figure 5 shows the ODF intensity of {110}<100> Goss, {110}<112> Brass, {110}<111> A, and {110}<110> Rotated Goss, which are the main texture components along the *α*-fiber in A700 and A800. Both specimens indicated high intensity of Brass, A component, and low-intensity Rotated Goss component, confirming that the specimens had similar textures.

**Figure 6:**
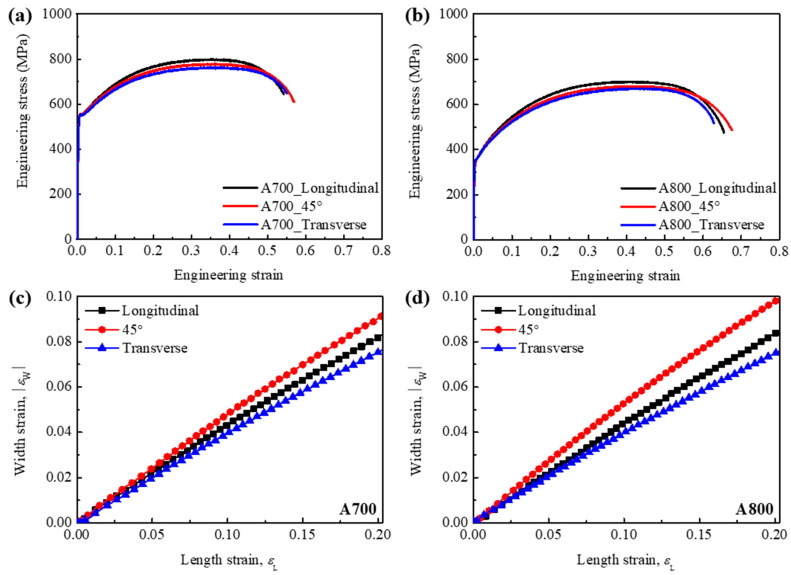
Engineering stress–strain curves of (**a**) A700 and (**b**) A800 specimens. Evolution of width strain (*ε*_W_) and length strain (*ε*_L_) during tensile loading along the different directions of (**c**) A700 and (**d**) A800 specimens.

These results are consistent with our experimental objectives mentioned above, and enable us to investigate the effect of second-phase precipitates (*σ* phases) on plastic anisotropy during plastic deformation. Figure 6a,b show the room-temperature tensile stress–strain curves of the A700 and A800 tensile specimens along three directions (longitudinal, transverse, and 45° directions). The results are presented in Table 2. The higher values of yield strength and tensile strength are obtained in the A700 specimens in comparison with those of the A800 specimens, while the elongation slightly decreases. A decrease in annealing temperature results in a decrease in grain size, thereby leading to the increase in the yield strength following the well-known Hall–Petch relationship *σ*_y_ *= σ*_0_ *+ k*_y_ *· d*^−1/2^ (*σ*_0_, frictional stress; *k*_y_, Hall–Petch coefficient; *d*, the average grain size). Furthermore, we note that the annealing at 700 °C leads to the formation of *σ* phase precipitates, which may also affect tensile properties due to the precipitation strengthening effects.

In both A700 and A800 specimens, on the other hand, the tensile properties vary with respect to the rolling direction. Figure 6c,d show the relationship between width strain (*ε*_W_) and length strain (*ε*_L_) during tensile loading along the different directions of the A700 and 800 specimens. During tensile loading, a relatively high *ε*_W_ is obtained along the 45° direction in both specimens, while the variation in *ε*_W_ along the three directions of the A800 specimen is larger than that of the A700 specimen. From these results, the *r* value along 0°, 45° and 90° to the rolling direction, $\overset{-}{r}$, and ∆*r* are also obtained, as summarized in Table 3. Since the *r* value is proportional to the *ε*_W_, the $\overset{-}{r}$ value of the A800 specimen, which indicates an extent of non-uniform plastic deformation, is much higher than that of the A700 specimen. On the other hand, The A700 specimen Is more planar Isotropic (∆*r* = −0.16) than the A800 specimen (∆*r* = −0.35). This planar anisotropy is known to affect the earing defect during the deep drawing process [27].

**Figure 7:**
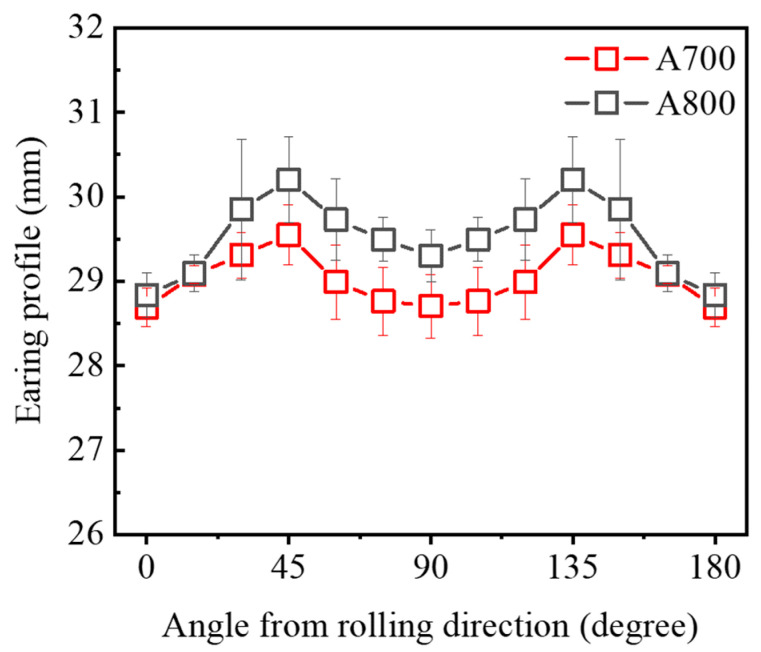
Earing profile representing the height of the cup specimens at drawing ratio of 2.0.

To thoroughly analyze the earing phenomenon, the heights of the deep-drawn cup specimens were measured along the circumferential direction from the rolling direction. Figure 7 displays the measured height profiles for the A700 and A800 cup specimens. At a specific stress level, the direction exhibiting high strain corresponds to a high *r*-value, forming a hill, and the opposite occurs in areas with lower strain. This earing is attributed to a non-zero ∆*r* value, which was measured during the tensile testing. Consequently, the ear heights in the A800 were much higher than those in the A700, resulting from a higher ∆*r* value in the A800 (−0.35) compared to the A700 (∆*r* = −0.16). The most notable aspect of these results is that A700 exhibits more planar isotropic behavior, even though the differences in constituent texture components between each specimen are negligible.

**Figure 8:**
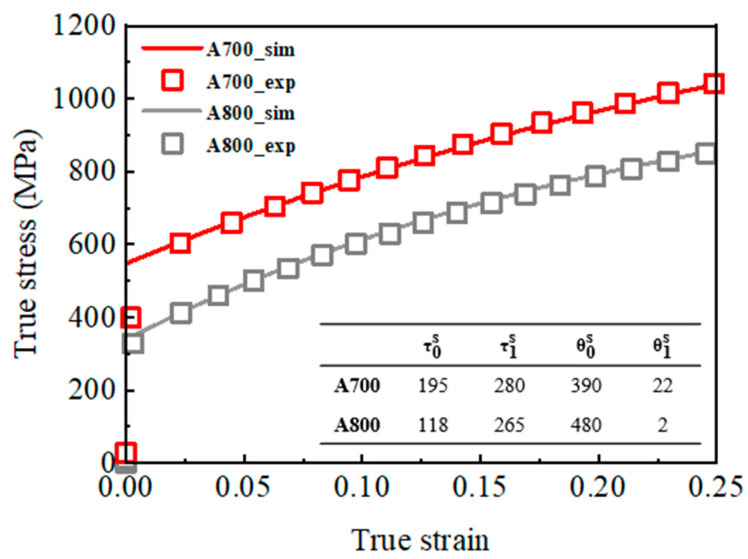
Experimental and simulated true stress–strain curves of the A700 and A800 specimens.

An in-plane anisotropy is primarily linked with a crystallographic texture. On the other hand, the texture components and their intensities constituted in the A700 and A800 are similar to each other. To investigate the effect of the crystallographic texture on the mitigation of plastic anisotropy in the A700 compared to the A800, we simulated a tensile test and obtained *r* values for the textures of the A700 and A800 specimens (Figure 4) with the VPSC simulations. The Voce hardening parameters, along with the stress–strain curves, are presented in Figure 8, and the simulated mechanical responses for both specimens are in good agreement with the measured ones, as shown in Figure 8. Although the A700 consists of *σ* phases and relatively finer grains in comparison to the A800, the crystallographic texture is mainly considered for simulating plastic anisotropy in the present VPSC model. This enables us to assess the texture-induced anisotropy. A comparison of the VPSC results with the corresponding experimental ones in Table 3 reveals that the predictions of the VPSC model are contradictory; the simulated ∆*r* for A700 and A800 are −0.43 and −0.45, respectively. We note that the present VPSC model did not take into account the full details of microstructural features such as the effect of precipitates, the evolution of in-grain misorientation, and the evolution of grain shape and annealing twins. Given that the constituent textures are mainly responsible for calculating the plastic anisotropy in the present VPSC model, differences in the anisotropy obtained from the simulations and experiments could be mainly attributed to the presence of precipitates. Indeed, the texture components and their intensities in A700 and A800 are similar to each other, and this may not lead to a significant difference in the plastic anisotropy calculated from the VPSC model. More specifically, since the A800 is composed of single-phase FCC solid solutions, the calculated *r* values of the A800 were reproduced satisfactorily compared to the experimental data, whereas the predicted *r* values for A700 contain large deviations. In this regard, further study utilizing comprehensive VPSC models that consider the Orowan equation, in which the plastic shear strain rate is related to dislocation density and velocity [28], is necessary to unravel the fundamental mechanism behind the effect of *σ*-phase formation on the slip activity and the resultant anisotropic behavior. 

Although few studies reported on the formability of HEAs, it has been recognized that the presence of non-shearable precipitates significantly affects the plastic anisotropy in heat-treatable aluminum alloys [27,29]. Engler et al. [30] investigated how the development of non-directional back-stresses around the non-shearable precipitates may affect the plastic anisotropy in Al7021 alloys. It is believed that the pile-up of dislocations around the precipitates is responsible for the formation of isotropic and texture-independent back-stresses, which mitigate the texture-induced planar anisotropy [30]. Jo et al. [10] reported that the *σ* phase acts as a non-shearable hard strengthener that hinders dislocation movement, contributing to the improvement of yield strength in VCrMnFeCoNi HEA. Such a feature was also reported in CoCrFeMnNi HEAs [11]. Therefore, it is plausible that the impediment of dislocation motion by the presence of *σ* phase may generate isotropic back-stresses around the precipitates, affecting the mitigation of the texture-induced anisotropy in A700.

## 4. Conclusions

The present work investigated the role of *σ*-phase precipitation on the plastic anisotropy of CoCrFeMnNi HEA. Annealing at 700 °C resulted in the formation of a relatively fine-grained microstructure with *σ* phase leading to higher strength, but similar texture components in comparison with the alloy annealed at 800 °C. The plastic anisotropy of the CoCrFeMnNi HEA annealed at 700 °C was two times lower than that of the one annealed at 800 °C. This is in line with the earing profiles of cup specimens after deep drawing. It was found that the alloy annealed at 700 °C is more planar isotropic in the sense of earing than the alloy annealed at 800 °C. The experimental results and VPSC models provided indirect evidence of the mitigation of plastic anisotropy by *σ*-phase precipitation. Overall, this study will open a new window to utilize the brittle sigma phase for optimizing mechanical properties and plastic isotropy of HEAs with great potential.

## Figures and Tables

**Table 1:** Chemical composition of FCC matrix and *σ*-phase in A700 specimen measured by TEM-EDS.

	Chemical Composition, at%				
Elements	Cr	Mn	Fe	Co	Ni
FCC matrix	19.21	20.12	19.06	20.77	20.84
*σ* precipitate	43.50	13.61	16.33	19.42	7.14

**Table 2:** Tensile properties of A700 and A800 specimens along the 0, 45, and 90 deg directions.

	A700			A800		
Degree	Yield Strength (MPa)	Tensile Strength (MPa)	Elongation (%)	Yield Strength (MPa)	Tensile Strength (MPa)	Elongation (%)
0°	540 ± 2	798 ± 4	54.2 ± 1.1	340 ± 4	701 ± 9	65.5 ± 0.8
45°	536 ± 1	778 ± 6	57.1 ± 1.6	333 ± 7	680 ± 7	67.5 ± 2.4
90°	530 ± 5	762 ± 5	55.1 ± 0.7	345 ± 8	671 ± 6	62.8 ± 1.3

**Table 3:** Plastic strain ratio (*r*-value), normal anisotropy ($\overset{-}{r}$), and planar anisotropy (∆*r*) obtained from experiments and VPSC modeling.

	A700		A800	
Parameters	EXP	VPSC	EXP	VPSC
*r*_0_	0.71 ± 0.02	0.75	0.69 ± 0.01	0.77
*r*_45_	0.83 ± 0.01	1.18	0.98 ± 0.03	1.22
*r*_90_	0.63 ± 0.03	0.60	0.58 ± 0.01	0.57
$\overset{-}{r}$	0.75	0.93	0.81	0.95
∆*r*	−0.16	−0.51	−0.35	−0.55

## Data Availability

Data are contained within the article.
